# Biological and clinical significance of iron in *Pseudomonas aeruginosa* biofilms

**DOI:** 10.1128/jb.00598-25

**Published:** 2026-05-28

**Authors:** Jacob M. Weiner, Rhishita Chourashi, Khady O. Ouattara, Amanda G. Oglesby

**Affiliations:** 1 Department of Pharmaceutical Sciences, University of Maryland, School of Pharmacy 12265, Baltimore, Maryland, USA; 2 Department of Microbiology and Immunology, University of Maryland, School of Medicine, Baltimore, Maryland, USA; Dartmouth College Geisel School of Medicine, Hanover, New Hampshire, USA

**Keywords:** *Pseudomonas aeruginosa*, biofilms, iron regulation, Fur, PrrF sRNAs

## Abstract

*Pseudomonas aeruginosa* is a ubiquitous gram-negative bacterium that causes diverse opportunistic infections in vulnerable populations. *P. aeruginosa* is innately resistant to many therapeutic agents, and biofilm-mediated infections demonstrate enhanced recalcitrance to antimicrobials. It has been known for over 25 years that iron plays a critical role in *P. aeruginosa* biofilm physiology, and numerous studies have revealed mechanistic insights into how this metallonutrient affects the regulatory, structural, and nutritional requirements of biofilm communities. This mini-review presents the current state of the field in understanding the specific impacts of iron on *P. aeruginosa* biofilm formation, and we propose key areas for future studies to more effectively relate these findings to the clinic.

## INTRODUCTION

*Pseudomonas aeruginosa* is a ubiquitous gram-negative bacterium that causes acute lung and blood infections in cancer patients and 10% of all hospital-acquired infections (1–4). *P. aeruginosa* also causes lifelong chronic lung infections in individuals with cystic fibrosis (CF) and is a significant contributor to chronic wound infections in diabetics and surgical patients (5–7). *P. aeruginosa* is innately resistant to many therapeutic agents, and the emergence of multi-drug-resistant (MDR) strains leads to persistent infections, longer hospital stays, and increased mortality rates (8, 9). Also problematic are biofilm-mediated infections, which exhibit increased tolerance to antimicrobials, nutrient starvation, and host immune assaults (10–12). Biofilms are aggregated and often surface-attached microbial communities that are encased in an extrapolymeric substance (EPS) consisting of a matrix of protein, extracellular DNA (eDNA), and polysaccharides (13–17). The EPS matrix, combined with slower bacterial growth and increased expression of multi-drug efflux pumps of biofilm cells, contributes to tolerance of biofilms to environmental assaults, including the host immune system and antibiotics (10, 12, 18).

Over 20 years ago, Singh and colleagues made the seminal discovery that lactoferrin, a component of the mammalian innate immune system that sequesters iron, disrupts *P. aeruginosa* biofilms (19). This finding has spurred numerous studies into the mechanisms by which this essential transition metal affects diverse mechanisms that are required for *P. aeruginosa* to shift from growth as single cells to the formation of a biofilm community. A recent review covered the impact of transition metals, including manganese, zinc, and iron, on biofilm formation by a range of microbial pathogens (20). This mini-review focuses on the current state of the field regarding the specific impacts of iron on *P. aeruginosa* biofilm formation, and we further discuss evolving therapeutic strategies aimed at disrupting iron homeostasis in *P. aeruginosa* biofilms.

## EXPERIMENTAL MODELS FOR BIOFILM STUDIES

*P. aeruginosa* biofilm infections include both device-mediated infections, which can be either acute or chronic, as well as chronic infections of the lungs, skin, and soft tissue. The former infections occur when *P. aeruginosa* colonizes a medical device—such as a catheter or ventilator—and forms a biofilm that can then disperse and spread to other organs (21–24). In contrast, chronic infections often occur due to underlying pathologies that promote microbial colonization and prevent clearance of the organism (7, 11, 21, 25, 26). A well-studied example of chronic *P. aeruginosa* infections is pulmonary infections in persons with the hereditary disease cystic fibrosis (CF). CF is characterized by the build-up of thick, sticky mucus in the lungs; eventual lung colonization by *P. aeruginosa* results in further decreased pulmonary function and eventual mortality (27–29). *P. aeruginosa* can also form antimicrobial-tolerant biofilms in wound beds, resulting in persistent inflammation, tissue damage, and slowed wound healing (7, 30, 31). One of the most problematic examples of these chronic wound infections are diabetic foot ulcers, which develop due to nerve damage and poor circulation; changes in vasculature, high blood glucose, and immune suppression of diabetic patients further complicate effective treatment of the ulcers (7, 32). As expected, the mechanisms of colonization, biofilm development, and environmental conditions vary considerably in each of these infections. Device-mediated infections occur after initial attachment to the plastic surface of a medical device (22, 33). In contrast, biofilms in the CF lung are often EPS-encased aggregates within mucus plugs (34), while biofilms in wound beds are attached to host cells and connective tissue (35). Additionally, the chronic CF lung is mildly acidic, largely hypoxic, and at body temperature (36, 37), whereas the wound bed environment is alkaline, at lower temperatures (33°C), and variable oxygen concentrations dependent on the depth of the wound bed (38, 39). Furthermore, *P. aeruginosa* strains that colonize the lungs of individuals with CF often overproduce the polysaccharide alginate (11), while small colony variants in the CF lung overexpress the Pel and Psl polysaccharides (40, 41). Biofilms formed in chronic wound infections rely more on the Pel and/or Psl polysaccharides (42).

A variety of *in vitro* biofilm models have been developed to study *P. aeruginosa* biofilms, each exhibiting strengths and limitations when relating findings to the diversity of *P. aeruginosa* biofilm infections (Table 1). *P. aeruginosa* biofilms easily form on both glass and plastic polymers that mimic hospital surfaces, contributing to nosocomial spread, as well as medical devices associated with *P. aeruginosa* infections (43). Macro-colony biofilms formed on agar have also been used to study cellular organization and spatial gene expression (44–48). Several media preparations that mimic the nutrient characteristics of different infections have also been developed. These preparations include wound-like media that capture prevalent factors encountered in damaged skin and soft tissue (49–51) and synthetic CF media (SCFM) preparations that mimic transcriptional profiles of *P. aeruginosa* colonizing the CF lung environment (52, 53). Furthermore, Moreau-Marquis et al. developed a tissue culture model wherein *P. aeruginosa* biofilms are formed on lung epithelial cells carrying a common CFTR mutation, revealing that CFTR cells secrete iron into the media to support *P. aeruginosa* biofilm formation (54, 55). *Ex vivo* models, including porcine bronchiolar tissue submerged in SCFM (56) and extracted CF sputum (57), have also been developed, allowing the introduction of additional host factors that are not as easily incorporated into *in vitro* models.

**TABLE 1:** *In vitro* and *ex vivo* biofilm models

Model	Type	Static or dynamic	Host factors	Model disease	Advantages	Disadvantages	References
Multiwell plates or culture tubes	*In vitro*	Static	No	N/A^*a*^	High-throughput screening of antibiotics and mutant strains.	Nutrient levels decrease over time. Accumulation of waste products and metabolites.	(55–57)
Macrocolony	*In vitro*	Static	No	N/A	Can view structural aspects and cellular arrangement.	Nutrient levels decrease over time. Accumulation of waste products and metabolites.	(41–45)
Drip flow reactor	*In vitro*	Dynamic	No	N/A	Nutrients are replenished and waste is removed. Reduced shear force.	Entire biofilm is not exposed to the same nutrient levels.	(58, 59)
Flow cell	*In vitro*	Dynamic	No	N/A	Nutrients are replenished and waste is removed. Entire biofilm exposed to consistent nutrient levels.	Increased shear force.	(19, 60–62)
Wound-like media	*In vitro*	Either	Yes	Chronic wound infection	Mimics factors found in damaged tissues.	Variable nutrient levels.	(46–48)
Cystic fibrosis epithelial cell cultures	*In vitro*	Either	Yes	Cystic fibrosis	Mimics the surface and host factors found in the host.	Does not mimic true *in vivo* environment.	(51, 52)
Ocular epithelial cell cultures	*In vitro*	Either	Yes	Keratitis	Mimics the surface and host factors found in the host.	Does not mimic true *in vivo* environment.	
Porcine lung/skin/cornea models	*Ex vivo*	Static	Yes	Cystic fibrosis, chronic wound, keratitis.	Mimics factors found in the host.	Does not mimic true *in vivo* environment.	(53)
Extracted cystic fibrosis sputum	*Ex vivo*	Static	Yes	Cystic fibrosis	Includes factors and nutrients directly from host CF lung environment	Does not mimic true *in vivo* environment.	(54)

^*a*^ N/A, not applicable.

One consideration for *in vitro* models is whether biofilms are formed in an open, dynamic system with a continuous supply of nutrients or in a closed, static system. Closed static models are widely used and are particularly beneficial for high-throughput screening of strains, environmental conditions, or antimicrobial compounds in multi-well plates (58, 60, 61). These models are inexpensive, simple, and reproducible, but they are characterized by the depletion of nutrients and accumulation of bacterial products that can alter biofilm growth and composition. In contrast, open biofilm systems provide a continuous supply of nutrients and removal of metabolites, which can control specific variables, such as iron. Peristaltic pumps can be used to control a continuous flow of media through narrow channels or flow cells, wherein biofilms form on an attached glass coverslip. Flow cell biofilms are most often grown at 25°C due to technical challenges with bubbles that form in the tubing as temperatures increase (63); however, our recent work demonstrates that with the right experimental setup, flow cell biofilms of *P. aeruginosa* can be reproducibly grown without disruption by bubbles at 37°C (64). In contrast, drip flow reactor (DFR) biofilms are grown with a constant flow of nutrients with the waste being removed by gravity, reducing sheer forces that can alter biofilm phenotypes in flow cell models. This DFR model was previously used to study *P. aeruginosa* metal homeostasis in response to calprotectin (CP), a host innate immune protein that sequesters multiple transition ions, including iron (65).

With the above considerations in mind, the choice of an *in vitro* model should be guided by the biological question being pursued experimentally. In the case of understanding the impact of iron homeostasis on biofilm physiology, iron concentrations can be precisely controlled over several days in flow cell systems, allowing investigators to better link mechanistic analysis of iron regulation with iron-dependent changes in biofilm physiology. In other cases, a static model may provide a more realistic model of certain biofilm infection sites, where nutrient limitation can develop over the course of biofilm growth due to host nutritional immunity (*vida infra*). However, it is important to note that immune activity due to tissue damage may also result in increased nutrient availability. The continued development of *in vitro* and *ex vivo* growth models that more closely mimic the host environment (e.g., tissues-on-a-chip) is needed to connect *in vitro* models to infection.

In this vein, several biofilm infection models have been developed to capture the full range of host-pathogen interactions that contribute to *P. aeruginosa* biofilm formation (Table 2). *P. aeruginosa* infects and forms biofilms in the model invertebrates *Drosophila melanogaster* and *Caenorhabditis elegans* (66–69), allowing the incorporation of understanding how host innate immunity affects biofilm development (70, 71). Larvae of the waxworm moth *Galleria mellonella* have also been used as an invertebrate infection model due to their low cost and ability to grow at 37°C (72–74). While *G. mellonella* infection recapitulates many phenotypic findings of *P. aeruginosa* infection in mammalian models, it remains unclear if *P. aeruginosa* similarly forms biofilms in this host. Moreover, as a non-model organism, there are fewer genetic tools to probe host-pathogen interactions in *G. mellonella* infections. Finally, several murine infection models have been developed to examine *P. aeruginosa* infection. Murine models include a CF lung infection model in which the epithelium sodium channel (ENaC) is over-expressed, resulting in similar lung pathologies as in CF (75), as well as chronic wound infection models where *P. aeruginosa* is inoculated into a skin incision (42, 76). Limited *in vivo* models have been applied to understand the role of iron in biofilm infection models. One of note used a slow killing (SK) *C. elegans* model, which is reliant on quorum sensing and results in biofilm growth in the nematode intestine, as well as a liquid killing (LK) model, which does not result in intestinal colonization and does not require quorum sensing (77). Analysis of *P. aeruginosa* mutants defective in the production of the pyoverdine siderophore revealed attenuation in the SK but not the LK model (77). Importantly, we recently found that the iron homeostasis requirements for biofilm formation are distinct at 37°C, which precludes *C. elegans* growth, as compared to 25°C (*vida supra*) (78).

**TABLE 2:** Animal models used to study biofilm infections

Animal model	Genetic manipulation	Model disease	Advantages	Disadvantages	References
*D. melanogaster*	Can be easily applied	N/A^*a*^	75% of human disease-related genes are conserved. Systemic immune response.	No adaptive immune system. Cannot be cultured at body temperature	(63, 64)
*C. elegans*	Can be easily applied	N/A	Transparency allows for imaging of live biofilms. Similar innate immune responses.	No adaptive immune system. Cannot be cultured at body temperature.	(65, 66)
EnaC mouse	Over expression of epithelium sodium channel	Cystic fibrosis	Spontaneous CF like disease. Similar immune response as human infection.	Short lifespan.	(72)
Chronic wound mouse	Can be easily applied	Chronic wound infection	Similar immune response as human infection. Genetically tractable to adapt to different underlying diseases.	Mouse skin and healing are structurally and physiologically different from human skin.	(39, 73)
Keratitis mouse	Can be easily applied	Keratitis	Similar immune response as human infection. Genetically tractable to adapt to different underlying diseases.	Limited physiological and anatomical similarity to humans	

^*a*^ N/A, not applicable.

Many chronic pseudomonal biofilm infections are polymicrobial, including those in wound beds and the lungs of individuals with CF (30, 32, 49, 79–83). Interactions between co-infecting pathogens result in increased antibiotic tolerance and virulence potential (84–86). Several reports have revealed that iron and nutritional immunity further impact the interactions of *P. aeruginosa* with other microbes (65, 87–89); however, the impact of iron on interspecies interactions within biofilms has yet to be explored. Recent work sought to determine the impact of incorporating commonly co-infecting organisms using the above-described models (49, 62, 76, 89–93). Although polymicrobial interactions significantly increase the complexity of these models, with the right controls, they will provide a better understanding of how iron affects microbial interactions in the context of polymicrobial infections.

## IRON AND NUTRITIONAL IMMUNITY

**Fig 1:**
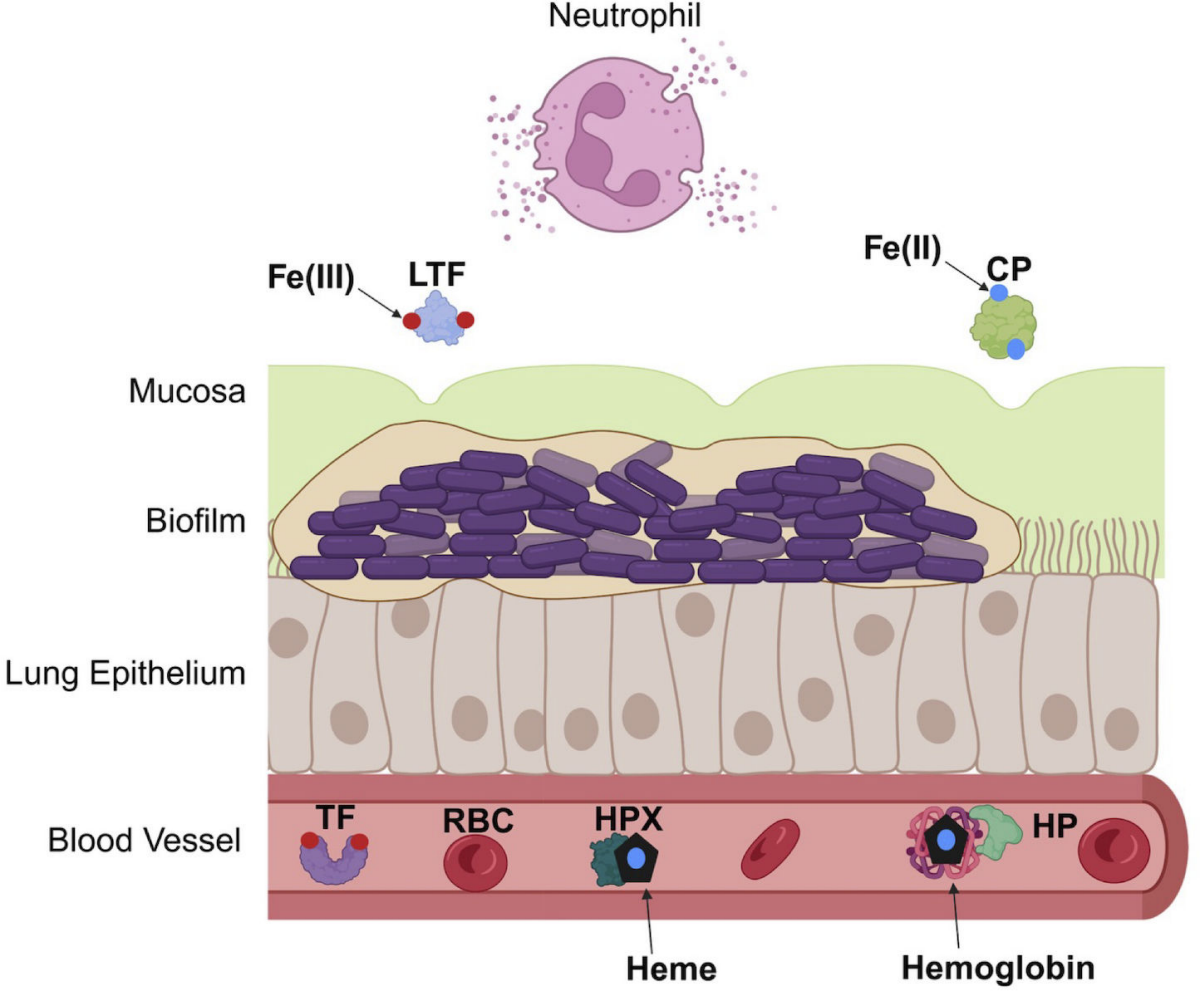
Human innate nutritional immunity proteins. Diagram of nutritional immunity proteins showing the Fe(III)-sequestering innate immune protein lactoferrin (LTF) and the Fe(II)-sequestering innate immune protein calprotectin (CP) being secreted by a neutrophil into the respiratory milieu at the site of a biofilm infection. Within the bloodstream, transferrin (TF) binds to Fe(III) to transport it between organs, and the hemopexin (HPX) and haptoglobin bind to heme and hemoglobin, respectively, that are released from dead red blood cells (RBC). Figure created in BioRender.

**Fig 2:**
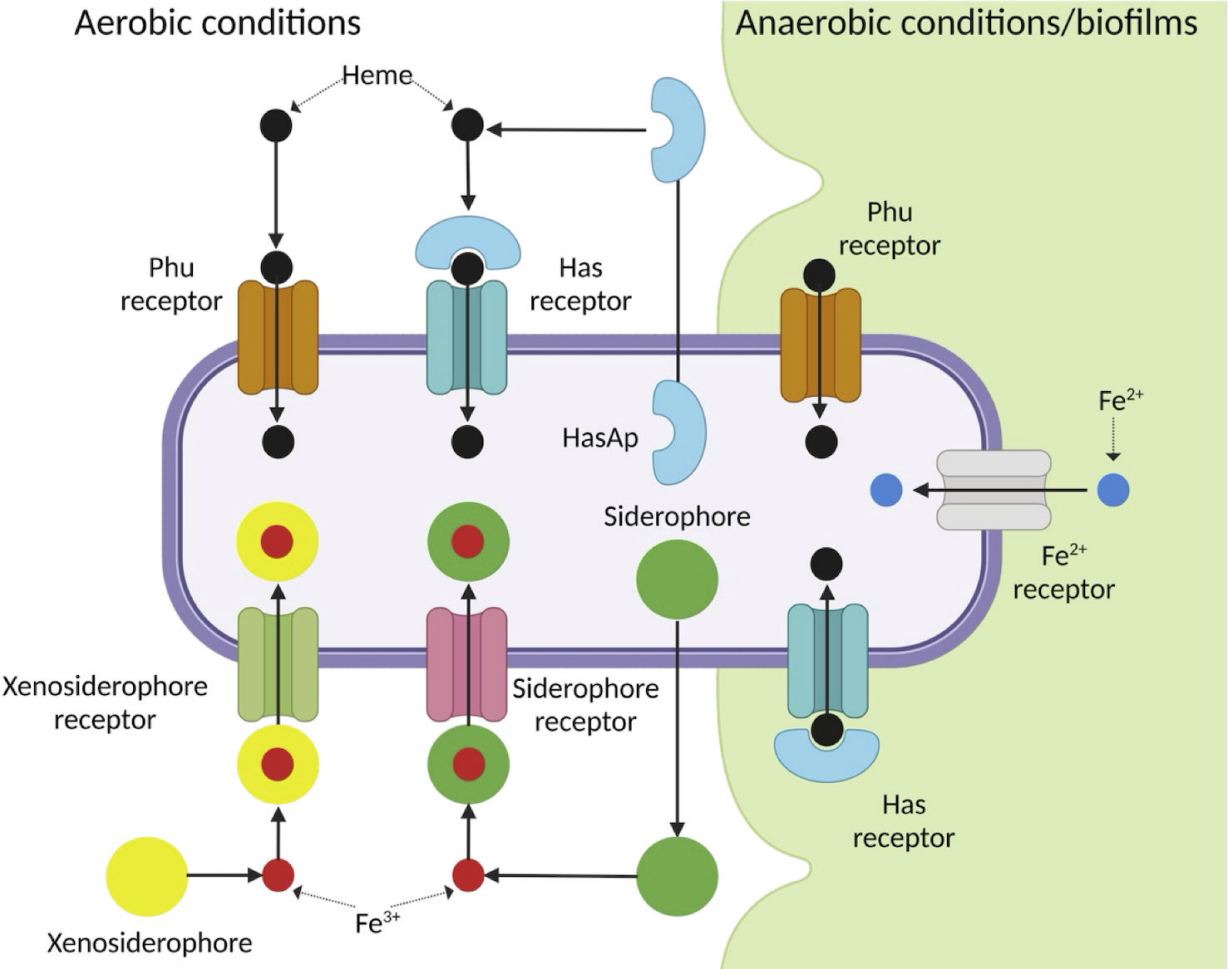
Iron acquisition by *P. aeruginosa*. Diagram of *P. aeruginosa* iron uptake strategies used in aerobic and anaerobic conditions. Under aerobic conditions, Fe(III) is scavenged through siderophore production (pyoverdine and pyochelin) followed by the uptake of ferri-siderophores. The genome of *P. aeruginosa* also encodes uptake systems for xenosiderophores that are produced by other microbes in the environment, allowing them to hijack the iron-scavenging systems of neighboring species. Under anaerobic conditions, where Fe(II) becomes the prevalent form of iron in the environment, *P. aeruginosa* predominantly acquires iron through the membrane-bound Fe(II) transport system (Feo). Additionally, heme can be utilized as an iron source under both aerobic and anaerobic conditions and is acquired through two nonredundant heme uptake systems. The *Pseudomonas* heme uptake system (Phu) uses a membrane-bound heme transporter to directly take up heme from the environment, while the heme acquisition system (Has) utilizes a secreted hemophore (HasAP) to scavenge for heme in the environment. The figure is created in BioRender.

Metals are critical for microbial growth and infection; thus, the host’s innate immune system sequesters transition metals, including iron, to prevent microbial growth in a process termed nutritional immunity (94). One of the best-studied iron sequestration strategies involves iron sequestration by host proteins, which are exemplified by lactoferrin that is present in mucosal secretions and binds with high affinity to the oxidized, ferric form of iron [Fe(III)] (Fig. 1) (95). Lactoferrin reduces biofilm growth of *P. aeruginosa* in a 25°C flow cell biofilm model, resulting in flat biofilms that lack the characteristic mushroom-shaped structure characteristic of *P. aeruginosa* biofilms (19). Lactoferrin also inhibits biofilm formation of *P. aeruginosa* clinical isolates grown in microtiter plates at 37°C (96). To overcome Fe(III) sequestration by the innate immune system, *P. aeruginosa* secretes two distinct siderophores, pyoverdine (PVD) and pyochelin (PCH), which sequester Fe(III) with high affinity to compete with lactoferrin and are required for full pathogenicity in several acute infection models (Fig. 2) (97–99). Flow cell biofilm studies conducted at 25°C with mutants defective for the production of each of these siderophores showed that PVD, but not PCH, is required for optimal biofilm formation (100). However, more recent studies indicate that PCH is more highly expressed at 37°C than at 25°C (101), raising the question of whether PCH may play a more important role in biofilm formation at higher temperatures.

Several studies have shown that siderophore production is reduced in *P. aeruginosa* strains isolated from the CF lung (102, 103), likely due to decreased oxygen availability that results in a higher prevalence of the reduced, ferrous form of iron [Fe(II)] (104). Consistent with this idea, we recently found that deletion of the genes for PVD and PCH production results in increased planktonic growth of *P. aeruginosa* grown in anaerobic conditions (14). In hypoxic environments, *P. aeruginosa* can acquire Fe(II) via the FeoAB transporter (Fig. 2) (105). To date, the innate immune protein calprotectin (CP) is the only known host factor that can sequester Fe(II) (Fig. 1) (106). CP is released by neutrophils at infection sites where it sequesters divalent transition metals, including Fe(II), and induces iron starvation responses in *P. aeruginosa* and other microbial pathogens (106–109). CP treatment results in reduced *P. aeruginosa* biofilm formation due to its metal-sequestering ability (65) and leads to the formation of a mesh-like structure of unknown composition around biofilms, which may enhance its ability to capture iron (90). Additionally, CP protects *Staphylococcus aureus* against the antimicrobial activity of *P. aeruginosa*, and these two pathogens are commonly co-isolated from CF lungs and wound beds (30, 32, 49, 79–83), during *in vitro* agar cultures, murine lung infection, and in the CF lung environment (65). A more recent study demonstrated that CP’s effect on *in vitro* co-cultures of these organisms is independent of its metal-sequestering function (110), suggesting broader roles for CP in modulating *P. aeruginosa* biofilm formation.

*P. aeruginosa* can also mediate the uptake and degradation of heme (14), a significant source of Fe in the human host (Fig. 2) (111). Heme is normally sequestered within cells but can be released upon tissue damage during infection (94). The host produces a protein called hemopexin to sequester released heme and transport it to the liver (Fig. 1) (112). Much less is known about the how heme and heme-sequestering immune functions affect *P. aeruginosa* biofilm formation. However, *P. aeruginosa* has been shown to evolve toward higher expression of heme uptake systems over the course of CF lung infection, suggesting the importance of this iron source in the CF lung (102, 103). In support of a role for heme in *P. aeruginosa* biofilm formation, a prior study demonstrated that synthetic inhibitors of the *P. aeruginosa* HemO heme oxygenase, which is required for *P. aeruginosa* to use heme as an iron source, protected *C. elegans* from *P. aeruginosa* killing (113). Understanding the interplay of this clinically relevant form of iron in *P. aeruginosa* biofilm biology is a critical question for the field to continue addressing.

## REGULATORY NETWORKS LINKING IRON AND BIOFILM FORMATION

Despite its necessity, iron also promotes the formation of reactive oxygen species that damage biomolecules, a process exacerbated by the production of oxidants from innate immune cells (114). *P. aeruginosa* must therefore balance its requirement for iron with strategies that limit its toxic potential. In iron-depleted environments, these strategies include the upregulation of iron acquisition systems and the downregulation of non-essential iron utilization pathways. As iron levels rise, cells must inversely affect the expression of these systems. The coordinated expression of these systems is mediated by the ferric uptake regulator (Fur) protein, which in iron-sufficient conditions binds to promoter regions of numerous genes encoding proteins for siderophore (PVD and PCH) synthesis and uptake, heme uptake and degradation, and the Feo Fe(II) transporter (115–117). Fur also represses expression of the PrrF small regulatory RNAs, which bind to and promote the degradation of mRNAs for non-essential iron-dependent pathways (118–120). In doing so, the PrrF sRNAs function as an Fe budgeter—sparing the use of Fe for only the most critical processes—when this nutrient becomes limiting. We previously showed that the PrrF sRNAs are expressed by clinical isolates of *Pa* from acute and chronic infections (121). Moreover, we detected high levels of the PrrF sRNAs in sputum isolated from individuals with CF, verifying that they are produced during chronic CF lung infection (102). Furthermore, the PrrF sRNAs are required for virulence in an acute lung infection model (121, 122). Combined, these studies highlight the intricate role of iron regulation in *P. aeruginosa* virulence.

**Fig 3:**
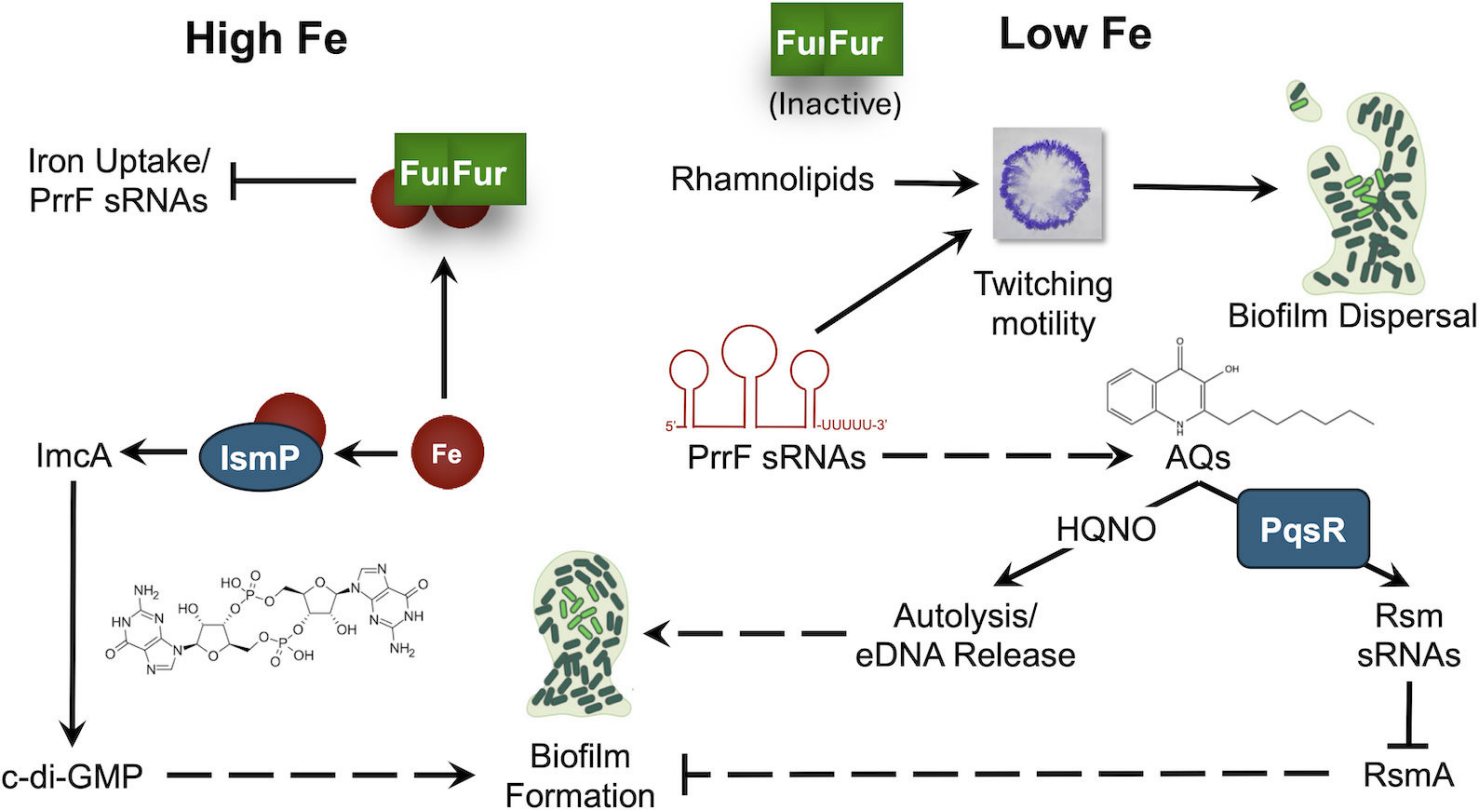
Iron regulatory pathways affecting *P. aeruginosa* biofilm formation. Solid lines indicate a [putative] direct effect. Dashed lines indicate indirect effects.

Previous findings show that inactivation of *fur* via point mutation allows *P. aeruginosa* to form its characteristic mushroom-shaped biofilms even in the presence of lactoferrin, while these structures are eliminated by lactoferrin treatment of the wild-type parent strain (100). In the same study, loss of the PrrF sRNAs had no impact on biofilm formation (100). Notably, both of these findings were made using flow-cell biofilms grown at room temperature, prompting our group to determine whether the same results would be obtained at body temperature. Importantly, we found that the PrrF sRNAs are indeed required for flow-cell biofilm formation of *P. aeruginosa* at 37°C but not at 25°C (78). While these studies demonstrate that iron regulatory pathways affect *P. aeruginosa* biofilm formation, it is unclear how Fur and PrrF specifically contribute to this process. Below, several models for how Fur and PrrF may affect *P. aeruginosa* biofilm formation are explored. An overview of these pathways is provided in Fig. 3.

### Quorum sensing

*P. aeruginosa* possesses three hierarchical quorum-sensing (QS) systems that mediate cell-to-cell signaling and play diverse roles in biofilm formation. Two of these systems rely on canonical N-acyl-homoserine-lactone (AHL) regulatory quorum-sensing molecules (Las and Rhl), while the third system is dependent on alkyl-4-quinolone (AQ) metabolites, including the PQS. The details of the interplay of the *P. aeruginosa* QS systems and regulatory controls are described elsewhere (123, 124). Here, we focus on studies implicating *P. aeruginosa* QS systems and how they contribute to iron-dependent biofilm formation. Earlier studies revealed that iron starvation results in full production of PQS molecules via regulation by the PrrF sRNAs (118, 120). This regulatory effect occurs via direct interaction of PrrF with the *antR* mRNA, encoding a transcriptional activator of genes for the conversion of anthranilate into TCA cycle intermediates. Anthranilate also serves as a precursor for PQS production, and PrrF repression of *antR* spares anthranilate for PQS production (118, 120). We recently showed that deletion of *pqsA*, encoding the initial gene for synthesis of PQS, results in a biofilm defect at 37°C but only in low iron conditions, essentially phenocopying the ∆*prrF* mutant (78). PqsA is also required for expression of the HSI-2 type VI secretion system involved in interbacterial interactions and biofilm formation in static cultures, which are likely more relevant to growth during chronic infection than shaking cultures (125). In strains that lack production of the Las QS molecule, PQS induces the production of the Rhl QS molecule by inducing the *rhl* genes under phosphate- and iron-starved conditions (126), a finding relevant to chronic infection of the CF lung, where the Las system is often lost (127). Detailed investigations of these regulatory pathways in clinical isolates and QS mutants that are relevant to chronic biofilm infections are needed to fully understand how these pathways affect biofilm formation during infection.

### Rsm system

The *P. aeruginosa* RsmY and RsmZ small RNAs play a central role in the switch from acute to chronic infection phenotypes. The Rsm sRNAs sequester RsmA, an RNA-binding protein that inhibits translation of various target genes in *P. aeruginosa* involved in biofilm formation (128–130). We recently established that RsmY and RsmZ sRNAs are induced by iron starvation, and this regulation occurs only in static conditions (131). This regulation is eliminated in a ∆*pqsA* mutant, further indicating the importance of PQS in chronic lifestyles such as biofilms. A third Rsm sRNA, RsmW, has also been identified to be upregulated during *P. aeruginosa* biofilm formation (132). While a few studies have examined the RsmW sRNA, published transcriptomic and proteomics analysis show that PA4570, a co-transcribed protein immediately upstream of the *rsmW* gene, is induced upon iron starvation (133, 134). Furthermore, a canonical Fur binding site is located in the promoter region of the *PA4570-rsmW* transcript. Altogether, these data suggest a role for Rsm involvement in iron-regulated biofilm formation by *P. aeruginosa.*

### Cyclic-di-GMP

Cyclic-di-GMP (c-di-GMP) is a second messenger that contributes to *P. aeruginosa’s* switch between planktonic and biofilm lifestyles (135)*. P. aeruginosa* exhibits increased c-di-GMP production during chronic infection, and this second messenger influences EPS production and other processes that promote biofilm formation through interactions with transcriptional activators (136)*,* the Rsm regulatory network (137), and EPS synthetic enzymes (138–140)*.* Cellular levels of c-di-GMP are controlled by diguanylate cyclases (DGC) and phosphodiesterases (PDE) that form and degrade c-di-GMP, respectively (135). Iron regulation was previously linked to c-di-GMP signaling in *P. aeruginosa* (141), although the precise nature of this link remains unknown. Our own laboratory’s work shows that induction of biofilm formation in *P. aeruginosa* by certain antibiotics, which is dependent on c-di-GMP signaling (142), is also dependent on iron and the PrrF sRNAs (121, 143). A recent report revealed that iron directly binds to an iron-sensing protein, IsmP, to inhibit its interaction with a DGC, ImcA (144); this binding results in decreased motility and increased biofilm formation. Cumulatively, these studies indicate that iron contributes to biofilm formation at least in part by controlling levels of c-di-GMP.

### Motility

*P. aeruginosa* possesses multiple surface appendages that play complementary roles in the initiation, development, and dispersal of biofilm communities. Two of these appendages, flagella and type IV pili (TFP), also contribute to surface-attached social motilities: twitching and swarming. Twitching motility is dependent upon the TFP (145), while swarming motility is dependent upon both flagella and TFP (146). In contrast to biofilm formation, iron has a negative impact on both types of social motility in *P. aeruginosa* (147–149), indicating that iron plays a key role in the “stay-or-go” decision by *P. aeruginosa* communities. Iron regulation of social motility is due in part to the induction of rhamnolipid production by iron starvation, resulting in enhanced twitching and swarming motility (149). The induction of rhamnolipid production by *P. aeruginosa* triggers biofilm dispersal (149), and the timing of rhamnolipid secretion affects biofilm architecture (59, 149). Our own laboratory showed that the PrrF sRNAs contribute to the induction of TFP protein production and twitching motility in iron-limiting conditions (134). Moreover, a previously published bioinformatic analysis using the CopraRNA tool identified complementarity between the PrrF sRNAs and the *pilQ* and *fimU* mRNAs (150), suggesting that PrrF regulation of twitching motility is due to a direct interaction of the PrrF sRNAs with TFP mRNA transcripts. Thus, iron regulation of *P. aeruginosa* biofilm formation may further be linked to iron-regulated changes in rhamnolipid production and motility.

## INFLUENCE OF IRON ON THE BIOFILM MATRIX

The EPS plays a key role in biofilm physiology and is dependent upon numerous factors, including the enzymes needed for continued maintenance of the matrix, eDNA that contributes to biofilm structure and horizontal gene transfer, and polysaccharides that aid in the adhesion, cohesion, and aggregation of the microbes (15). The primary polysaccharides of the *P. aeruginosa* EPS are Pel, Psl, and alginate, each providing distinct contributions to the attachment and structural stability of the biofilm matrix. These contributions vary in different conditions and strains; hence, we refer the reader to more detailed reviews of these polysaccharides and their contribution to the biofilm matrix (17, 151, 152). Studies have shown that iron represses expression of the AmrZ transcriptional regulator, which, in turn, promotes production of the Psl polysaccharide that itself sequesters and stores iron (153). In contrast, iron limitation increases the production of alginate in laboratory strains, while not affecting alginate production in mucoid CF isolates (154). How each of these regulatory effects coordinately affect biofilm formation, structure, and stability during infection remains unknown.

A prior study from Yang et al*.* showed that iron limitation promotes cell death and eDNA release (via a process now referred to as “explosive cell lysis” [155]) to promote *P. aeruginosa* biofilm formation (156), a seemingly paradoxical finding given that iron limitation is known to reduce biofilm formation (19). In the same study, enhanced eDNA release and biofilm formation in low iron conditions were dependent on *pqsA* as well as *pqsR*, the latter encoding the response regulatory system for PQS formation (156). In contrast, deletion of *pqsL*, which is required for the synthesis of HQNO via the AQ biosynthetic pathway (157), led to increased programmed cell death, eDNA release, and biofilm formation in low iron conditions (156). This finding is distinct from a more recent study showing that deletion of *pqsL* results in reduced eDNA production and biofilm formation, indicating that HQNO promotes rather than prevents these activities (158). Importantly, the latter study was performed using LB, which contains approximately 40 µM iron, a concentration identified by inductively-coupled plasma mass spectrometry (ICP-MS) (Nguyen and Oglesby, unpublished data), while the former study was performed using a minimal medium (AB) supplemented with specific concentrations of iron. Moreover, the earlier study employed *P. aeruginosa* strain PAO1, while the latter study used strain PA14. Strain difference is an important variable to consider, given that each of these *P. aeruginosa* strains exhibits distinct methods of biofilm initiation correlated with different c-di-GMP signaling effects (159). More work is therefore needed to elaborate the role of iron in eDNA release and determine how this affects *P. aeruginosa* biofilm formation and structure, especially from strain to strain and in clinical isolates.

Outer membrane vesicles (OMVs), which carry various gram-negative bacterial cargo, including secondary metabolites, nucleic acids, and proteins, have also been implicated as important components of bacterial biofilm matrices (recently reviewed in reference 160). Production of OMVs is promoted by PQS and facilitates cell-to-cell transfer of PQS and other hydrophobic QS molecules (161). The presence of OMVs within the matrices of *P. aeruginosa* biofilms was first demonstrated in 2006 by Schooling and Beveridge using a variety of biofilm models, including agar colony, flow-cell biofilms, and DFR (162). Since then, *P. aeruginosa* OMVs have been shown to promote either the formation or dispersal of biofilms, depending on the source of the OMVs (163–165). One of these studies showed that PQS contributes to OMV-mediated biofilm dispersal in a manner independent of its QS activities (164). Several studies have further shown that the cargo of *P. aeruginosa* OMVs varies depending on the growth conditions of cells producing the OMVs (162, 165). The PrrF sRNAs may therefore impact the production of OMVs and biofilm architecture in low iron conditions via its positive impact on PQS production (118, 120).

## IRON-BASED THERAPEUTIC STRATEGIES

Due to *P. aeruginosa’s* requirement for iron during biofilm formation and infection, disrupting *P. aeruginosa* iron homeostasis has been a long-considered strategy for novel antimicrobial development. In one example, two FDA-approved Fe(III) chelators—desferoxamine and desferasirox—enhanced the ability of the aminoglycoside antibiotic tobramycin to clear *P. aeruginosa* biofilms on CF cell lines (54). Tobramycin is commonly administered to individuals with CF to help control *P. aeruginosa* colonization, but its efficacy decreases as chronic biofilm-containing infections worsen (166, 167). Our laboratory’s own studies further demonstrated how loss of PVD siderophore production can enhance the efficacy of tobramycin against *P. aeruginosa* biofilms grown as colonies on agar plates or in microtiter plates (143). These studies provide proof-of-principle that disruption of iron homeostasis could prolong the efficacy of current antimicrobial therapies.

In line with these studies, many researchers have worked toward the development of a “Trojan Horse” antibiotic that is conjugated to a siderophore functional group, allowing the antibiotic to be taken up through siderophore receptors and overcoming issues with cell permeability (168). One such antibiotic, cefiderocol, was approved in 2019 for treatment of complicated urinary tract infections, when caused by gram-negative bacteria including *P. aeruginosa* (169), and for treatment of hospital-acquired and ventilator-associated pneumonias that are recalcitrant to standard therapies (169). Cefiderocol remains a last-line treatment in these cases due to poor outcomes reported in the CREDIBLE-CR trial, which showed increased morbidity in some patients where it was used (170). This has been attributed in part to heteroresistance to cephalosporins, wherein portions of the clinical strain population exhibit resistance to the antibiotic that is not easily detected in the clinical laboratory (171). Cefiderocol is also unlikely to be effective for treatment of CF airway infections due to non-siderophore iron acquisition strategies becoming more prominent in these individuals (*vida infra*) (102, 103). Nonetheless, cefiderocol provides an example of the potential broad success of therapeutic strategies targeting *P. aeruginosa* iron homeostasis. Additional strategies in the pre-clinical stage include the targeting of the heme oxygenase required for using heme as an iron source (113, 172, 173) and blocking the mobilization of iron from bacterioferritin, representing the primary iron storage protein in *P. aeruginosa* (174).

Researchers have also studied the effects of gallium treatment due to its chemical similarities with iron, including comparable ionic radii, electron affinity, electronegativity, and coordination number. Gallium-68 (^68^Ga) is FDA-approved for use as a radiotracer during positron emission tomography (PET) scans, and gallium nitrate (GaNO_3_, marketed as Ganite) is an FDA-approved treatment for hypercalcemia. Prior work showed that GaNO_3_ disrupts *P. aeruginosa* iron metabolism and inhibits biofilm formation (175). Further research has examined the antimicrobial activities of different gallium-containing compounds, such as gallium nitrate (175, 176) and gallium maltolate (177), as well as heme mimics gallium-protoporphyrin (178) and gallium-salophen (172). Ganite was previously examined in a phase 2 clinical trial for efficacy against *P. aeruginosa* in pwCF (NCT02354859), and an ongoing phase 2 trial is investigating this treatment in pwCF who are colonized with non-tuberculosis *Mycobacteria* (NTM, NCT04294043), highlighting the potential efficacy of these therapeutics against chronic *P. aeruginosa* infections in the CF lung.

Additionally, recent studies have also looked at leveraging the host nutritional immunity proteins as novel therapeutics. An example of this is the use of the Fe(II) chelator calprotectin to treat *P. aeruginosa* biofilms (179, 180). Calprotectin alone has been shown to inhibit the growth of *P. aeruginosa* in murine wound models (65) and enhances biofilm clearing when treated in combination with current antibiotics such as ciprofloxacin (180). Another example is the use of the Fe(III) chelator lactoferrin, which has been shown to inhibit the growth of *P. aeruginosa* biofilms by triggering bacterial motility. When lactoferrin is combined with the rare sugar alcohol xylitol, *P. aeruginosa*’s ability to respond to the damage induced by lactoferrin-mediated iron chelation is inhibited (181). Furthermore, a novel lactoferrin-conjugated gallium complex has been developed and has been shown to have antimicrobial activity against *P. aeruginosa* biofilms (182). As our understanding of how iron homeostasis influences biofilm growth increases, there will be more emphasis on developing iron-focused therapeutics.

## FUTURE INVESTIGATIONS

Biofilm infections by *P. aeruginosa* are substantial contributors to public health, and iron homeostasis has been established as a central mediator of both infectivity and biofilm formation by this pathogen. The past 25 years have established many converging roles for iron in *P. aeruginosa* biofilm physiology, outlined in this review. Future studies in this area should be directed toward not only a deeper understanding of the mechanisms underlying phenomena described in this review but also toward increasing the clinical relevance of these findings. While mammalian models are invaluable tools for identifying and validating potential drug targets, robust *in vitro* models that incorporate clinically-relevant environmental variables are indispensable for defining mechanisms that can lead to drug development. We propose the following directions as critical next steps to bridge current iron and biofilm studies to the clinic.

### Temperature-dependent iron homeostasis

Temperature is a key factor for virulence gene regulation in many pathogens; however, limited studies have addressed how temperature affects *P. aeruginosa* gene regulation (101, 183). *P. aeruginosa* is the only well-studied pseudomonad that both grows at body temperature and can cause infection, suggesting that growth at 37°C was an evolutionary step toward becoming an opportunistic pathogen. Two independent studies have shown that *P. aeruginosa* significantly changes in its transcriptome when grown at 22°C vs. 37°C, including a shift from PVD to PCH siderophore gene expression at 37°C, suggesting that iron homeostasis plays a key role in adaptation to body temperature (101, 183). Additional studies have revealed that temperature alters *P. aeruginosa* phage gene expression, as well as the matrix composition in *P. aeruginosa* biofilms (184, 185). Temperature varies between specific infection environments, with wound infections experiencing temperatures around 33°C, while blood and organ infections occur at 37°C. Moreover, environmental or room temperature (25°C) is highly relevant to *P. aeruginosa* reservoirs in clinical settings, which contribute to nosocomial infections. A fundamental understanding of how temperature affects molecular processes underlying *P. aeruginosa* iron homeostasis in biofilms is therefore crucial to develop novel infection control and therapeutic strategies.

### Adaptations by clinical isolates

While laboratory strains provide robust tools for genetic analysis, clinical isolates of *P. aeruginosa* readily adapt to the host environment. Moreover, the genome of *P. aeruginosa* exhibits high plasticity, and laboratory-adapted strains vary between research groups (186–188). Thus, it is critical to assess clinical isolates with limited passaging to determine the relevance of findings from laboratory strains. Multiple resources exist for obtaining such isolates, including the CF Isolate Core at Seattle Children’s Hospital, which houses decades of clinical isolates of *P. aeruginosa* and other pathogens from children and adults with CF. It is also important to consider the impact of clinical advances that have altered the underlying pathologies associated with earlier studies of *P. aeruginosa* clinical isolates. For example, CFTR modulator therapies and improved antimicrobial regimens have greatly reduced pulmonary disease in persons with CF (pwCF), resulting in significant shifts in the etiology of CF lung infections (189–191).

### Polymicrobial interactions

Chronic biofilm infections are often polymicrobial in nature, and interspecies interactions can dramatically alter the physiology and virulence capacity of each colonizing organism (49, 51, 76, 91, 92, 192–195). For example, the presence of *S. aureus* enhances virulence factor production, including AQs by *P. aeruginosa* (91). Furthermore, our own work has shown that iron limitation enhances killing of *S. aureus* by *P. aeruginosa* by enhancing the production of AQs (87, 88), and prior work revealed that killing of *S. aureus* provides an iron source to *P. aeruginosa* (196). *P. aeruginosa* siderophores have also been implicated in the ability of *P. aeruginosa* to compete with other microbes, including in biofilms (197). Curiously, the introduction of calprotectin to *P. aeruginosa/S. aureus* cocultures result in reduced *P. aeruginosa* AQ production and increased *S. aureus* survival during coculture (65), despite the ability of this innate immune protein to elicit iron starvation responses in both organisms (107, 109). However, our recent collaborative work has discovered that the effects of calprotectin on *P. aeruginosa/S. aureus* coculture dynamics occur via a distinct metal-independent manner that includes decreased AQ production by CP-treated *P. aeruginosa* (110, 198). *S. aureus* has further been shown to modify and inactivate PCH, and *S. aureus* mutants lacking this enzymatic activity are less competitive in a murine wound infection model (199, 200). Recent work developed a representative polymicrobial model for CF lung infection by growing mixed cultures of *P. aeruginosa*, *S. aureus*, *Streptococcus sanguinis*, and *Prevotella melaninogenica* in an artificial sputum medium containing mucin (192, 201). Initial work with this model demonstrated that loss of LasR, which is common in CF isolates (127), resulted in increased tolerance of *P. aeruginosa* to tobramycin in these mixed communities (192). Subsequent studies with this model revealed metabolic cross-feeding, wherein *P. aeruginosa* metabolism of short-chain fatty acids supported the growth of *P. melaninogenica*, which cannot survive in the medium during monoculture (201). Understanding how such interspecies interactions affect iron homeostasis pathways of each organism in such a community is vital for determining the clinical relevance of currently published work.

### Role of nutritional immunity factors

The underlying basis for studying iron homeostasis in microbial pathogens is the central role of this nutrient in host-pathogen interactions. However, metal limitation in laboratory media while also recapitulating the host environment can be technically challenging. One issue is the introduction of host-relevant factors such as mucin into *in vitro* systems, as these factors are often contaminated with metals that can complicate metal homeostasis studies. This issue can be overcome through the introduction of host-relevant iron sequestration factors such as lactoferrin and calprotectin. Indeed, the initial study that led to the body of work in this review used lactoferrin to demonstrate the importance of iron in *P. aeruginosa* biofilm formation (19). Another issue is whether *P. aeruginosa* and other relevant pathogens can survive without supplementation of iron and other transition metals under certain clinically relevant conditions, as this can hamper careful metal regulation studies. In our recent work, we found that *P. aeruginosa* would not grow anaerobically without iron supplementation; however, we were able to recapitulate iron starvation effects through treatment of anaerobic cultures with CP, revealing substantial impacts of oxygen on *P. aeruginosa* iron homeostasis pathways (14). Additional studies with CP have further revealed the effects of this important innate immune factor that are independent of metal sequestration (65, 108, 110, 202), yielding new directions for investigating how *P. aeruginosa* interacts with the host and co-colonizing pathogens during chronic biofilm infections.

## SUMMARY

Iron homeostasis is a central determinant of *P. aeruginosa* biofilm physiology and pathogenicity. Over the past two and a half decades, research has revealed how iron availability influences *P. aeruginosa* regulatory networks, extracellular matrix composition, and interactions with host immunity and co-infecting organisms. Moving forward, research should prioritize further development of clinically relevant models to bridge mechanistic findings of *P. aeruginosa* iron homeostasis pathways with patient outcomes. These efforts will help guide the development of iron-focused therapeutic strategies that are effective for the treatment of chronic biofilm infections involving *P. aeruginosa*.
